# Ablation of the miRNA cluster 24 in cartilage and osteoblasts impairs bone remodeling

**DOI:** 10.1038/s41598-022-13231-z

**Published:** 2022-06-01

**Authors:** Veronika S. Georgieva, Björn Bluhm, Kristina Probst, Mengjie Zhu, Juliane Heilig, Anja Niehoff, Bent Brachvogel

**Affiliations:** 1 Center for Biochemistry, Faculty of Medicine, University of Cologne, 50931 Cologne, Germany; 2 Department of Pediatrics and Adolescent Medicine, Experimental Neonatology, Faculty of Medicine, University of Cologne, 50931 Cologne, Germany; 3 Institute of Biomechanics and Orthopaedics, German Sport University Cologne, 50933 Cologne, Germany; 4 Cologne Center for Musculoskeletal Biomechanics (CCMB), University of Cologne, 50931 Cologne, Germany

**Keywords:** Bone remodelling, Cartilage development

## Abstract

MicroRNAs (miRNAs) post-transcriptionally regulate cartilage and bone development and function, however, only few miRNAs have been described to play a role for cartilage to bone transition in vivo. Previously, we showed that cartilage-specific deletion of the Mirc24 cluster in newborn male mice leads to impaired growth plate cartilage development due to increased RAF/MEK/ERK signaling and affects the stability of the cartilage extracellular matrix on account of decreased SOX6 and SOX9 and increased MMP13 levels. Here, we studied how Mirc24 cluster inactivation in cartilage and osteoblasts leads to an increased bone density associated with defects in collagen remodeling in trabecular bone. No changes in osteoblast distribution were observed, whereas the number of osteoclasts was reduced and TRAP activity in osteoclasts decreased. Surprisingly, an increased level of cluster-encoded miR-322 or miR-503 raises *Rankl* gene expression and inactivation of the cluster in chondrocytes reduces *Rankl* expression. These results suggest that the Mirc24 cluster regulates *Rankl* expression in chondrocytes at the chondro-osseous border, where the cluster is mainly expressed to modulate osteoclast formation, bone remodeling and bone integrity.

## Introduction

MicroRNAs (miRNAs) are key post-transcriptional regulators of cell proliferation, differentiation and cell death in a wide range of tissues. Nevertheless, their role for cartilage-mediated bone formation and remodeling is still not completely understood. These small non-coding RNAs can regulate the expression of several mRNA targets simultaneously and thereby modulate the activation of signaling pathways and diverse cellular functions. Recent studies showed that miRNAs regulate chondrocyte differentiation and function in vitro^1^, but only few miRNAs have been described to play a role for skeletal development and cartilage to bone transition in vivo.

The transient growth plate cartilage provides a scaffold for the formation of the endochondral skeletal elements and directs their longitudinal growth. It is gradually replaced by bone in the process of endochondral ossification. Chondrocytes within the growth plate proliferate and differentiate into prehypertrophic and hypertrophic cells that lay down a mineralized cartilaginous extracellular matrix (ECM) and initiate bone formation. Hypertrophic cells undergo apoptosis or transdifferentiate into osteoblasts^2–4^ as blood vessels invade cartilage and bring in chondroclasts, osteoclasts and osteoblasts, which remove the ossified cartilage matrix at the chondro-osseous junction and replace it with bone matrix^5^. The process of endochondral ossification is highly organized and strictly regulated by transcription factors (SOX9, RUNX2), growth factors (BMPs, TGFβs, FGFs) and their downstream effectors (SMADs, MAPKs)^6^. The small non-coding miRNA molecules add another level of complexity to the strictly organized process of cartilage and bone development.

The role of miRNAs for endochondral ossification was first demonstrated by the cartilage-specific deletion of the miRNA-processing enzymes Dicer and Drosha in mice. The genetic ablation of Dicer caused an overall reduction of miRNAs and led to a severe skeletal dysplasia and premature death due to tracheal collapse and respiratory failure^7,8^. Cartilage-specific deletion of Drosha had a similar effect leading to decreased miRNA levels, reduced chondrocyte proliferation, impaired skeletal development and perinatal death of the animals^9^. Specifically, let-7 miRNAs and miR-140 were shown to regulate chondrocyte proliferation and differentiation and, while the single knockouts presented with a mild skeletal phenotype, deletion of both miRNAs in mice markedly reduced skeletal growth^10^. Furthermore, miRNAs were shown to play a role for osteoblast and osteoclast formation by conditional deletion of Dicer. Ablation of Dicer in osteoprogenitors impaired osteoblast maturation and matrix mineralization and caused embryonal lethality^11^, whereas its conditional deletion in osteoclasts decreased osteoclast numbers, suppressed bone resorption and increased bone mass^12^. These studies demonstrate that miRNAs play a crucial role in skeletal development.

Previously we found the miRNA cluster Mirc24 to be predominately expressed in musculoskeletal tissues. This cluster encodes for miR-322, miR-351 and miR-503, but in cartilage only miR-322 and miR-503 are increasingly expressed in prehypertrophic and hypertrophic chondrocytes. We recently used Col2a1-Cre mice^13^ and floxed *Mirc24*^*tm1M/tm1M*^ mice^14^ to produce hemizygous Col2a1-Cre-*Mirc24*^*tm1M/Y*^ males with a full cartilage-specific deletion of the Mirc24 locus on the single X-chromosome and a genetic ablation of the locus in cartilage, which leads to the activation of the RAF/MEK/ERK pathway and impaired cartilage development^15^. Transgenic hemizygous Col2a1-Cre-*Mirc24*^*tm1M/Y*^ newborn males have a decrease of the hypertrophic area of the growth plate and a reduction in the diameter of tracheal cartilage rings. The decrease in tracheal diameter causes perinatal death due to respiratory failure and the analysis of the cartilage-specific deletion of the Mirc24 locus for cartilage homeostasis was focused on newborn mice. Mirc24 cluster deficiency in chondrocytes decreased SOX6 and SOX9 protein levels and altered the abundance of several proteins of the extracellular matrix (ECM), suggesting that cluster-encoded miRNAs fine tune ECM production and degradation in cartilage^16^. Interestingly, bone density is increased in newborn Mirc24 cluster-deficient mice^15^, but the underlying pathomechanism remains to be elucidated. Here we studied the consequences of Mirc24 cluster inactivation in cartilage for bone formation and remodeling in Col2a1-Cre-*Mirc24*^*tm1M*^ newborn male mice.

## Results

### Loss of the Mirc24 cluster in cartilage leads to an increase of bone density

**Figure 1:**
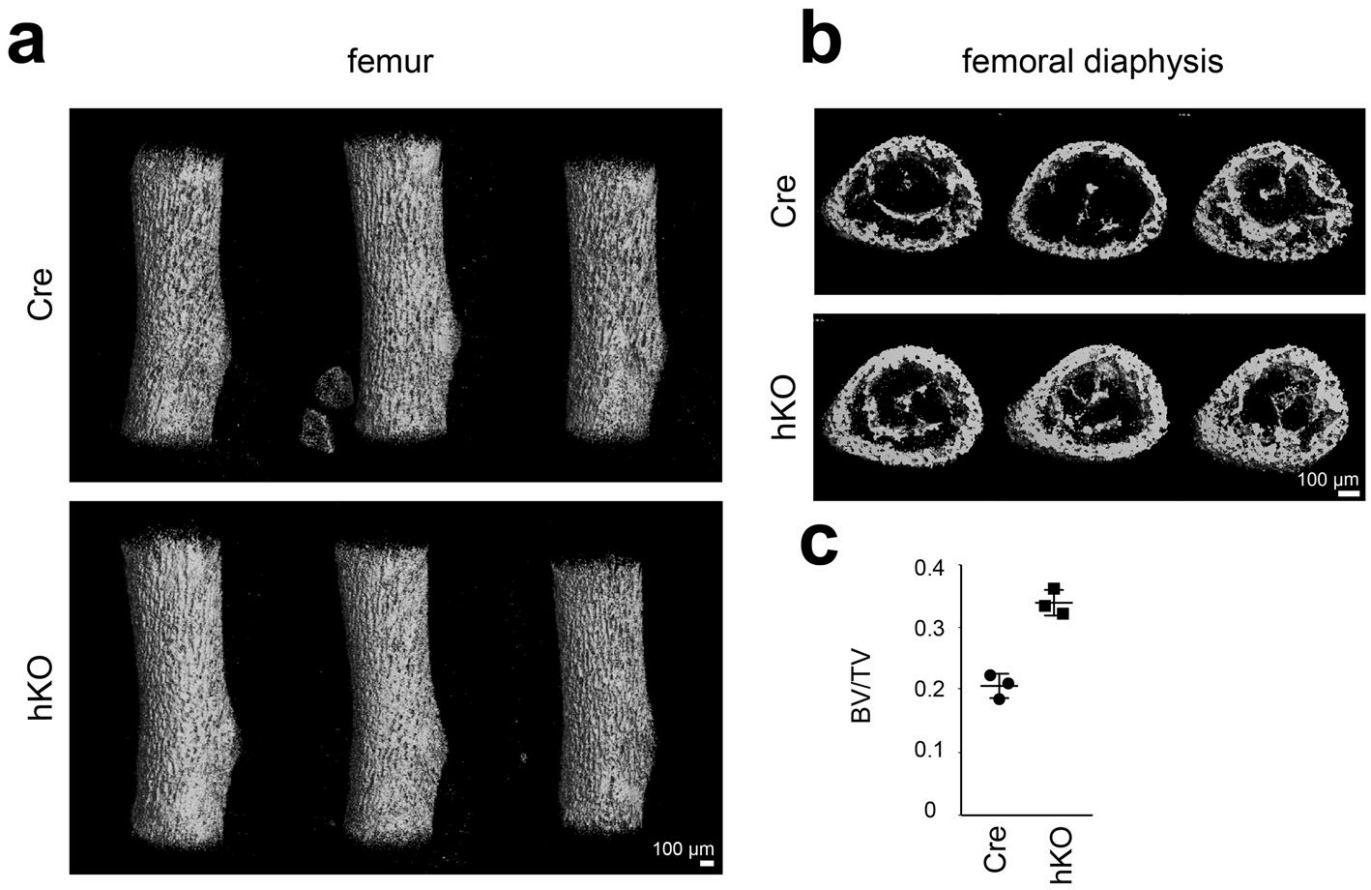
Analysis of femora of Mirc24 cluster-deficient mice. μCT analysis of femora from newborn male Col2a1-Cre (Cre) and Col2a1-Cre-*Mirc24*^*tm1M/Y*^ (hKO) mice. Three animals per genotype are shown. (**a**) Three dimensional reconstructions of the femora. (**b**) Cross-sections of the femoral diaphysis. (**c**) Relative bone volume (BV/TV). Individual and mean values and standard deviations are shown (graph). The figure was created with Adobe Illustrator CS6 (Version 16.0.3, https://adobe.com/products/illustrator).

Previously we observed an increase of bone density of vertebral bodies in newborn male Col2a1-Cre-Mirc24^*tm1M/Y*^ (hemizygous, hKO) mice compared to Col2a1-Cre (Cre) littermates^15^. Further analysis by micro CT (μCT) now revealed an increase of bone density (see Fig. 1a) and thickness of cortical bone of isolated femora in hKO mice (see Fig. 1b). Quantification indicated a 1.6-fold increase in relative bone volume (bone volume/tissue volume) in hKO mice (see Fig. 1c).

**Figure 2:**
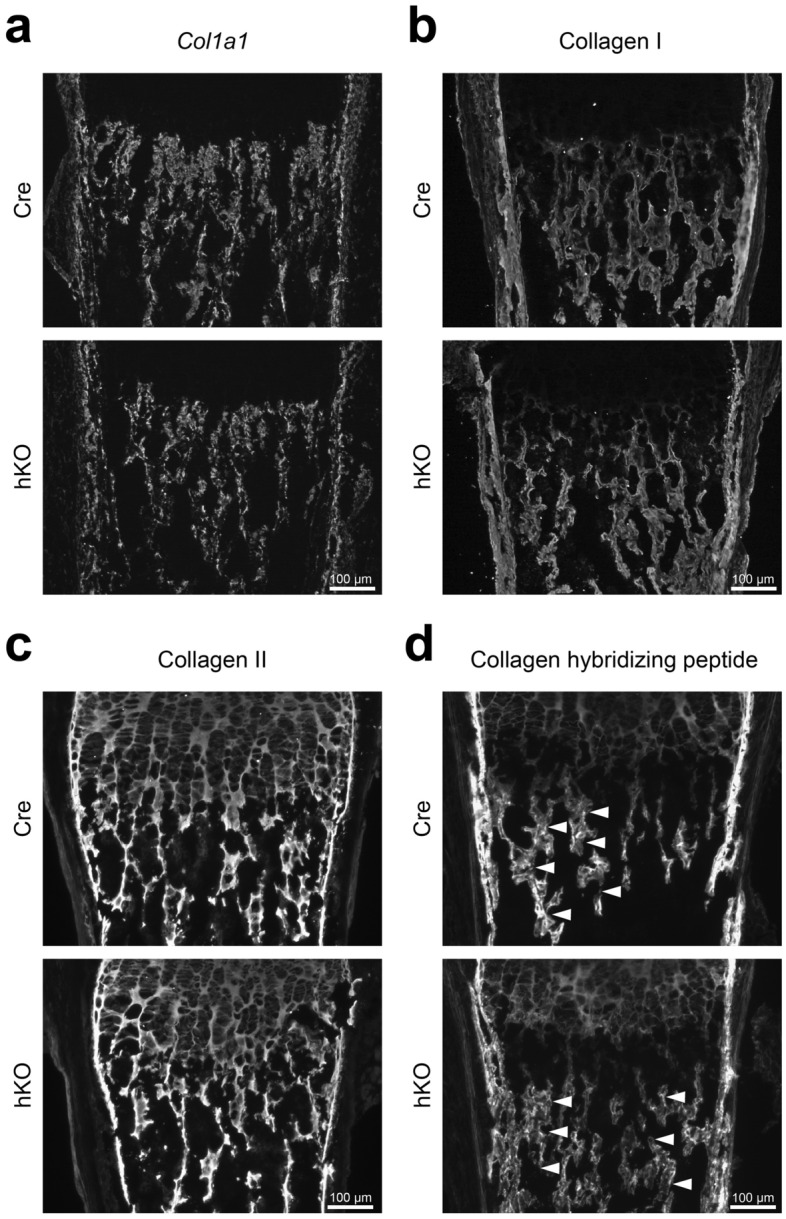
Analysis of the abundance of fibrillar collagens in femoral sections of Mirc24 cluster-deficient mice. Detection was performed on plane matched sections of femora from newborn male Col2a1-Cre (Cre) and Col2a1-Cre-*Mirc24*^*tm1M/Y*^ (hKO) mice. (**a**) *Col1a1* mRNA distribution was analyzed by fluorescence in situ hybridization using a *Col1a1* specific fluorescence probe. The abundance of (**b**) collagen I and (**c**) collagen II was studied by immunofluorescence. (**d**) In addition, to study bone remodeling the binding of collagen hybridizing peptide to unfolded collagen chains was analyzed by fluorescence microscopy. Note reduced intensity of the trabecular staining (arrowheads). Brightness was adjusted for visualization (**a**–**c**) using Adobe Photoshop Elements 2020 (Version 18.0, https://www.adobe.com/products/photoshop-elements.html). Four animals per genotype were analyzed and representative images are shown. Additional images are shown in Supplementary Figs. S1 and S2 online. The figure was created using Adobe Illustrator CS6 (Version 16.0.3, https://adobe.com/products/illustrator).

We hypothesized that the alterations in bone density may be accompanied by denser collagen fibers and therefore analyzed the deposition of the fibrillar collagens of bone and cartilage, collagen I and collagen II, in frozen femoral sections of newborn male mice with a cartilage-specific genetic ablation of the Mirc24 cluster. *Col1a1* gene expression was analyzed by fluorescence in situ hybridization and a strong signal was detected in the periosteum, as well as in cortical and trabecular bone (see Fig. 2a and Supplementary Fig. S1 online). In addition, collagen I protein distribution was studied by immunofluorescence staining. Here collagen I protein was detected in the bony structures of the femora (see Fig. 2b and Supplementary Fig. S1 online) and no changes in distribution and intensity of the collagen I gene expression or protein deposition were seen between genotypes. Next, collagen II protein distribution was studied. Collagen II was found throughout the different zones of the cartilage growth plate, in the perichondrium and in the remnants of calcified cartilage in the bone marrow cavity, as previously described^17^ (see Fig. 2c and Supplementary Fig. S2 online), but no alterations were observed between genotypes. The remodeling of the trabecular collagen matrix in bone was then analyzed by detecting the interaction of a Cy3-labled synthetic peptide (collagen hybridizing peptide, CHP) with degraded, unfolded collagen chains using fluorescence microscopy^18^. A fluorescent signal was observed throughout the cartilage growth plate in the region of collagen II expression. In addition, a strong fluorescent signal was detected in the periosteum, cortical and trabecular bone of Cre mice. In hKO mice the intensity of the trabecular staining was reduced, whereas the growth plate staining remained unchanged (see Fig. 2d and Supplementary Fig. S2 online). These results indicate that the loss of the Mirc24 cluster in cartilage and osteoblasts is linked to an aberrant remodeling of fibrillar collagens in the bone marrow matrix.

### Osteoclast numbers are reduced in Mirc24 cluster-deficient mice

**Figure 3:**
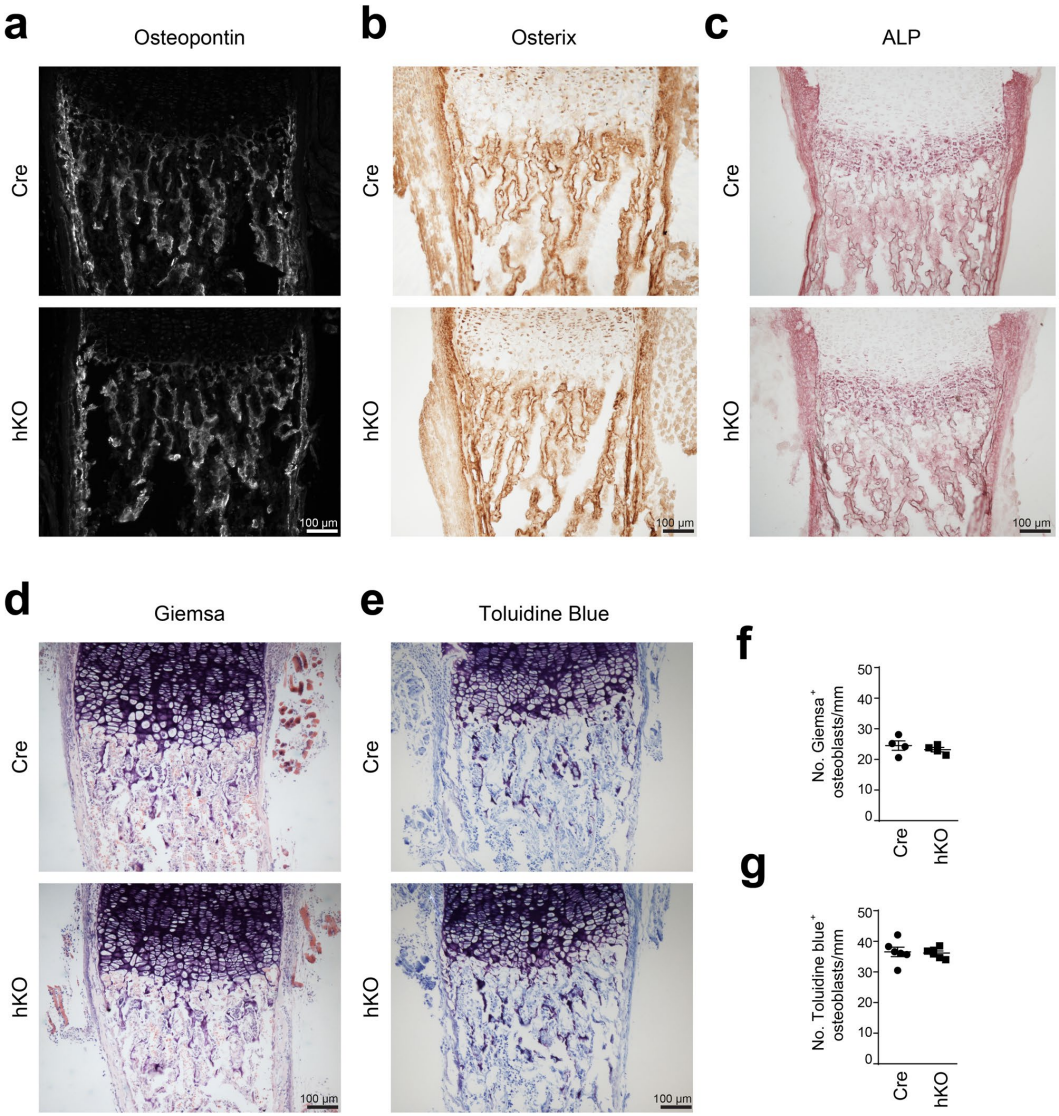
Characterization of osteoblast organization in femoral sections from Mirc24 cluster-deficient mice. Osteoblast distribution in plane matched sections of femora from newborn male Col2a1-Cre (Cre) and Col2a1-Cre-*Mirc24*^*tm1M/Y*^ (hKO) mice was analyzed by immunodetection of (**a**) osteopontin and (**b**) osterix. (**c**) In addition, alkaline phosphatase (ALP) activity was detected by histological staining. (**d**,**e**) To determine osteoblast numbers, paraffin sections were stained with Giemsa and Toluidine Blue. Brightness was adjusted using Adobe Photoshop Elements 2020 (Version 18.0, https://www.adobe.com/products/photoshop-elements.html) for visualization (**d**,**e**). At least three animals per genotype were analyzed and representative images are shown. Additional images are shown in Supplementary Figs. S3 and S4 online. (**f**,**g**) For quantification, the number of Giemsa^+^ and Toluidine Blue^+^ osteoblasts per mm trabecular bone were determined. Individual and mean values and standard deviations are shown (graph). The figure was created using Adobe Illustrator CS6 (Version 16.0.3, https://adobe.com/products/illustrator).

The decrease in collagen remodeling and bone density could be caused by an imbalance between cartilage-mediated bone formation and resorption and we therefore determined osteoblast and osteoclast distribution in femoral sections of newborn male mice with cartilage-specific genetic ablation of the Mirc24 cluster. Osteoblast distribution was analyzed by immunodetection of osteopontin (OPN) and osterix in Cre and hKO mice. OPN deposition was detected in late hypertrophic chondrocytes, as well as in trabecular and cortical bone in accordance with previous reports^19^. No differences in distribution and intensity of the OPN staining were seen between genotypes (see Fig. 3a and Supplementary Fig. S3 online). Osterix localization was analyzed by immunohistochemical staining and a strong signal was observed in trabecular and cortical bone (see Fig. 3b and Supplementary Fig. S4 online). Osterix was also expressed in the perichondrium and protein levels were not altered in hKO mice. Osteoblast-specific activity of alkaline phosphatase (ALP), as an early marker of osteoblast differentiation, was determined by histological staining. Phosphatase activity was detected in the perichondral and trabecular bone, as well as in late hypertrophic chondrocytes and some variations were observed between the genotypes, but no significant differences were found (see Fig. 3c and Supplementary Fig. S4 online). Finally, the number of osteoblasts in close association with the trabecular network of the bone marrow were quantified using Giemsa and Toluidine Blue staining. Here, no differences in osteoblast numbers were observed (see Fig. 3d–g).

**Figure 4:**
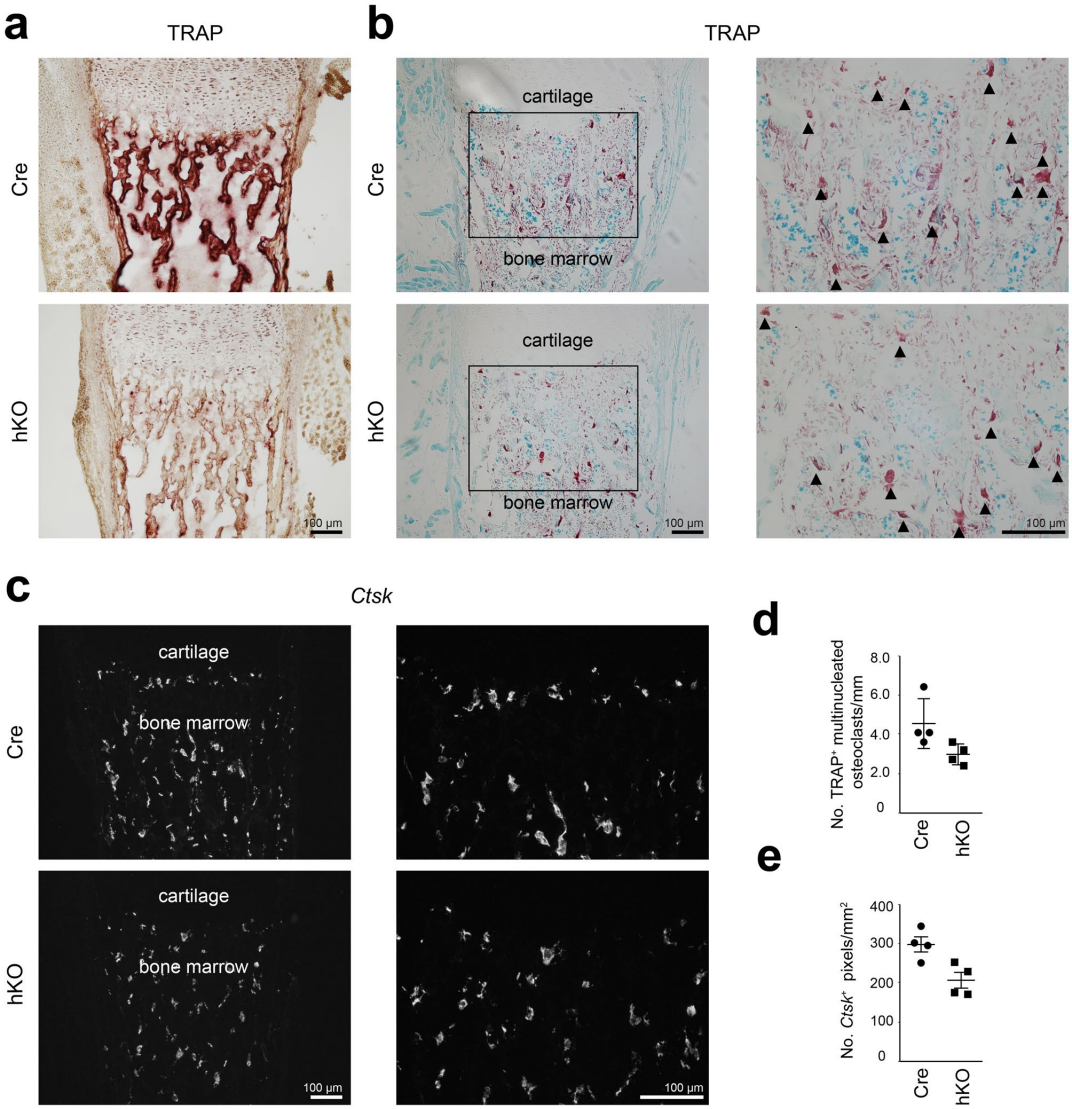
Characterization of osteoclast organization in femoral sections from Mirc24 cluster-deficient mice. (**a**) To identify osteoclasts, tartrate-resistant acid phosphatase (TRAP) activity stainings were performed on frozen sections of femora from newborn male Col2a1-Cre (Cre) and Col2a1-Cre-*Mirc24*^*tm1M/Y*^ (hKO) mice. (**b**) To determine osteoclast numbers, TRAP activity (red) was detected in paraffin sections counterstained by Fast green (blue). Enlarged area is indicated by a box. Multinucleated osteoclasts are marked by arrowheads. (**c**) *Ctsk* mRNA distribution was analyzed by fluorescence in situ hybridization using a *Ctsk* specific fluorescence probe. Brightness was adjusted for visualization (**b**,**c**) using Adobe Photoshop Elements 2020 (Version 18.0, https://www.adobe.com/products/photoshop-elements.html). Four animals per genotype were analyzed and representative images are shown. Additional images are shown Supplementary Figs. S5 and S6 online. For quantification (**d**), the number of TRAP^+^ multinucleated osteoclasts per mm trabecular bone in (**b**) was determined and (**e**) the number of *Ctsk*^+^ particles per mm^2^ in (**c**) was calculated. Individual and mean values and standard deviations are shown (graph). The figure was created using Adobe Illustrator CS6 (Version 16.0.3, https://adobe.com/products/illustrator).

Next, osteoclasts were studied by detecting the tartrate-resistant acid phosphatase (TRAP) activity. TRAP is produced and secreted by osteoclasts from the ruffled border area to modulate osteoclast migration^20,21^. A strong TRAP activity was detected at the chondro-osseous junctions and along the trabecular bone in frozen sections of Cre mice and activity was strongly reduced in hKO animals (see Fig. 4a and Supplementary Fig. S5 online). An optimized TRAP activity staining procedure^22^ was then applied to paraffin sections to determine osteoclast numbers (see Fig. 4b and Supplementary Fig. S5 online). Here, individual TRAP^+^ osteoclasts were detected in the trabecular bone, as well as at the endo-osseous and chondro-osseous junction. Interestingly, the number of TRAP^+^ multinucleated cells per mm trabecular bone was significantly reduced by 1.5-fold in hKO animals compared to Cre control (P = 0.028, see Fig. 4d). In addition, the gene expression of *Ctsk*, encoding for the cysteine protease cathepsin K (CTSK), was analyzed by fluorescence in situ hybridization. CTSK is a potent collagenase degrading collagen I and collagen II, which is predominantly secreted by osteoclasts and synovial fibroblasts^23^. A *Ctsk*^+^ signal was detected in cortical and trabecular bone of Cre mice and signal intensity was reduced in hKO animals (see Fig. 4c and Supplementary Fig. S6 online). The number of *Ctsk*^+^ pixels per mm^2^ was reduced by 1.4-fold in hKO animals compared to Cre control (see Fig. 4e). The results show that loss of the Mirc24 cluster-encoded miRNAs in cartilage reduces osteoclast numbers and TRAP activity in the cartilage-derived bone marrow cavity.

### Loss of the Mirc24 cluster in cartilage reduces *Rankl* gene expression in chondrocytes

Receptor activator of nuclear factor-kappaB ligand (RANKL) induces osteoclast differentiation by binding to its receptor RANK on the surface of osteoclast precursors, whereas osteoprotegerin (OPG) binds to RANKL to prevent its actions and protect bone from excessive resorption. Both cytokines are synthesized by hypertrophic and prehypertrophic chondrocytes at the site of Mirc24 cluster expression to regulate osteoclast formation in the bone marrow^24^. We hypothesized that the lack of Mirc24 cluster-encoded miR-322 and miR-503 could inhibit RANKL or stimulate OPG expression to impair osteoclast formation and bone remodeling at the chondro-osseous junction. Therefore, *Rankl* and *Opg* gene expression was characterized in primary epiphyseal chondrocytes (PECs) after increasing cluster-encoded miRNA levels or in hKO mice.

**Figure 5:**
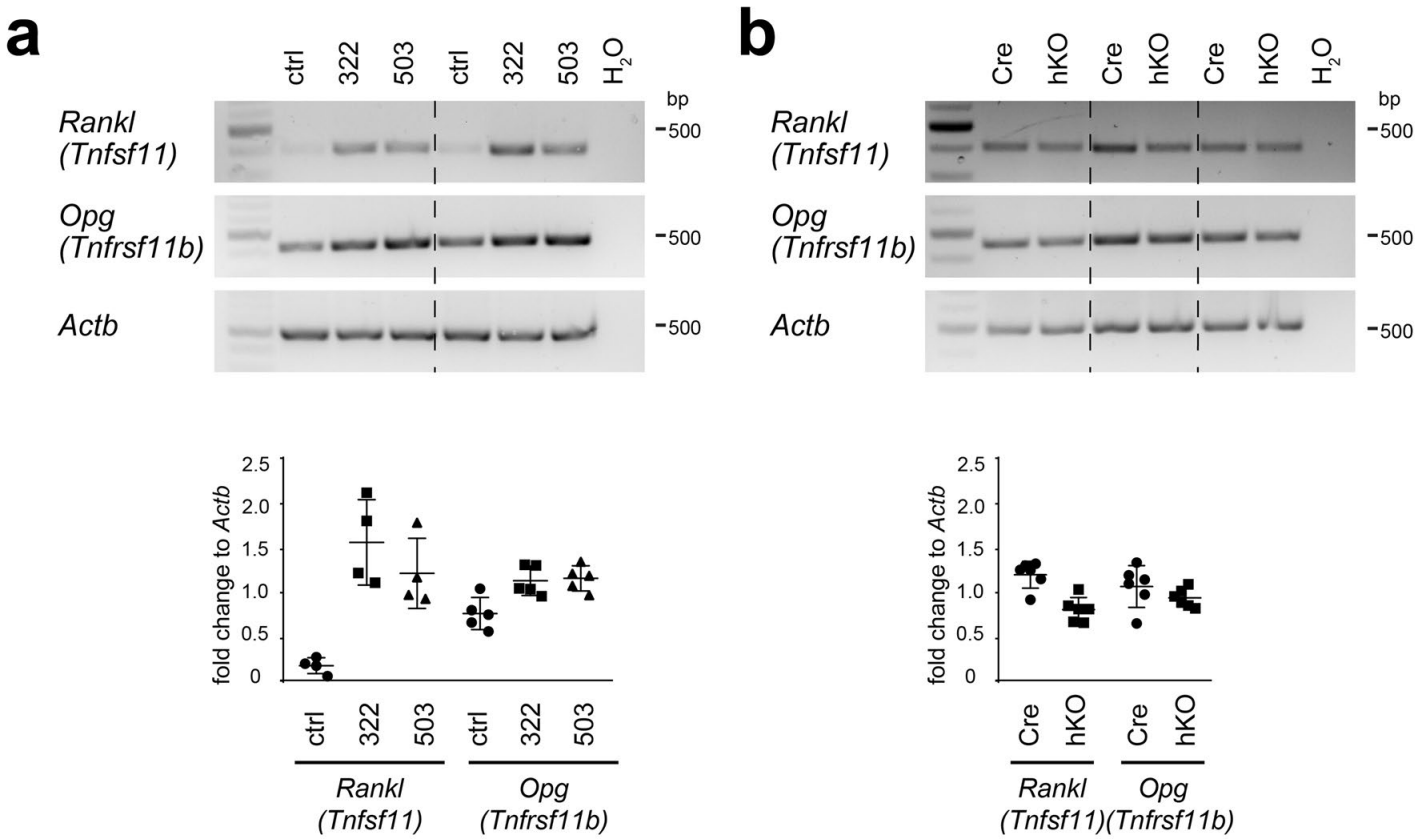
Determination of *Rankl* and *Opg* gene expression in miRNA mimic-transfected chondrocytes and in cultured chondrocytes from Mirc24 cluster-deficient mice. The gene expression of *Rankl* and *Opg* was analyzed by semi-quantitative PCR (**a**) in control (ctrl), miR-322 (322) or miR-503 (503) mimic-transfected primary epiphyseal chondrocytes (PECs) 2 days post transfection and (**b**) in PECs isolated from newborn male Col2a1-Cre (Cre) and Col2a1-Cre-*Mirc24*^*tm1M/Y*^ (hKO) mice and cultured for 2 days. The control (H_2_O) contains no genomic DNA and *Actb* served as loading control. *Rankl* or *Opg* to *Actb* ratio was determined by ImageJ and individual and mean values and standard deviations are shown (graph). The size of NEB Quick-Load Purple 1 kb Plus DNA Ladder bands is shown. The figure was created using Adobe Illustrator CS6 (Version 16.0.3, https://adobe.com/products/illustrator).

Gene expression of *Tnfsf11* and *Tnfrsf11b*, encoding for RANKL and OPG, respectively, was analyzed by semi-quantitative PCR in control- and miRNA mimic-transfected chondrocytes 2 days after transfection (see Fig. 5a). Relative expression levels were normalized to β-actin (*Actb*) and quantified by ImageJ. A significant 8- and 6-fold increase in *Rankl* mRNA (P = 0.021) was detected upon transfection of miR-322 and miR-503, respectively, as well as a 2.5-fold increase in *Opg* (P = 0.028 and 0.016). Next, *Rankl* and *Opg* expression was determined in chondrocytes isolated from newborn Cre and hKO mice and cultured for 2 days. *Rankl* was significantly decreased 1.5-fold in hKO PECs (P = 0.006), whereas *Opg* mRNA levels remained unchanged (see Fig. 5b). Hence, *Rankl* and *Opg* gene expression is upregulated in the presence of increased miR-322 and miR-503 levels, whereas absence of the miRNAs leads to decreased *Rankl* expression in chondrocytes. These data suggest that osteoclastogenesis is reduced in chondrocytes lacking the Mirc24 cluster, probably due to lower RANKL expression by chondrocytes at the chondro-osseous junction.

## Discussion

Although there have been many studies on endochondral ossification, it is still poorly understood how chondrocytes transduce signals to osteoclasts to induce their differentiation and trigger bone resorption. Previously, we showed that the Mirc24 cluster is expressed in the prehypertrophic and hypertrophic zone of growth plate cartilage^15^, as well as in articular cartilage^16^ and plays a role in cartilage development and ECM organization. In addition, we observed increased bone density of vertebral bodies^15^ and femora of newborn male Mirc24 cluster-deficient mice (see Fig. 1). Since miR-322 and miR-503 are the only Mirc24-encoded miRNAs expressed in cartilage, the effects of cluster inactivation in cartilage could be attributed to these two miRNAs. Here, we examined the consequences of Mirc24 cluster genetic ablation in cartilage for bone formation and remodeling and show that this cluster plays a role in chondrocyte-induced osteoclastogenesis.

We hypothesized that Mirc24 cluster-encoded miRNAs may modulate the balance of osteoclast and osteoblast numbers and activity to regulate bone density and this was supported by histological analysis of femora of newborn Mirc24 mice. A balance between osteoblast and osteoclast activity is crucial for maintaining bone mass and homeostasis and preventing osteoporosis or osteopetrosis^25^. Mirc24 cluster-encoded miRNAs have already been shown to directly regulate osteoblast and osteoclast differentiation. MiR-322 stimulates osteoblast differentiation in vitro by increasing the expression of osteogenic genes like *Sp7* (osterix), *Msx2* and *Runx2*^26^, whereas miR-503 inhibits RANKL-induced osteoclast formation from peripheral blood mononuclear cells in vitro by directly targeting *Rank*^27^. Nevertheless, the role of miR-322 and miR-503 expression in cartilage for the chondrocyte-dependent formation of osteoblasts and osteoclasts has not been described. Col2a1-Cre mediated recombination results mainly in a deletion of the cluster in chondrocytes, but also in a subpopulation of osteo-chondroprogenitor cells^28,29^, in osteoblast-derived from hypertrophic chondrocytes^2,3^ or skeletal progenitor cells originating from dedifferentiated hypertrophic chondrocytes^4^. Interestingly, we could confirm that a deletion of the cluster is detected in bone and that its expression is reduced in isolated osteoblasts (see Supplementary Fig. S8 online). However, we could not detect changes in the number of osteoblasts, in the localization and distribution of the osteoblast markers osteopontin and osterix by immunostainings or in the osteoblast activity by alkaline phosphatase staining (see Fig. 3). In contrast, reduced osteoclast activity was observed in Mirc24-deficient mice in a histological TRAP staining. The activity of this enzyme was strongly reduced at the chondro-osseous junction and along the trabecular bone of hKO animals (see Fig. 4a,b), due to lower numbers of osteoclasts is these animals (see Fig. 4d). The reduced number of osteoclasts was accompanied by a lower gene expression of *Ctsk* (see Fig. 4c,e). Osteoclasts secrete CTSK and hydrochloric acid to the resorption lacunae to degrade the extracellular matrix and hydroxyapatite, respectively, and less degradation can lead to a denser bone matrix. Collagen is extensively remodeled during embryonic bone formation and CTSK cleaves the triple helices of both collagen I and collagen II^30^. While *Col1a1* expression and the distribution of collagen I and II were not altered (see Fig. 2a–c), we could observe reduced collagen remodeling in the trabecular area of Mirc24 cluster-deficient mice (see Fig. 2d), probably due to lower *Ctsk* expression. Hence, loss of the cluster in cartilage and osteoblasts mainly reduces osteoclast numbers and activity and may directly impair bone resorption and remodeling.

The cluster may modulate osteoclast numbers and activity by secreting soluble factors at the chondro-osseous junction. Originally, bone marrow stromal cells/osteoblasts were considered as the main cell populations contributing to osteoclast differentiation by the expression of the cytokines RANKL and OPG. Recently, a growing number of studies have shown that prehypertrophic and hypertrophic chondrocytes also express RANKL and OPG and are the essential source of RANKL during bone mineralization^31^. We hypothesized that Mirc24 cluster-encoded miR-322 and miR-503 are increasingly expressed in the prehypertrophic/hypertrophic zone of growth plate cartilage to stimulate RANKL or decrease OPG expression and induce osteoclast formation and bone remodeling at the chondro-osseous junction. Our results show that while miR-322 and miR-503 transfection in chondrocytes increases mRNA levels of *Rankl* and *Opg* (see Fig. 5a), absence of the Mirc24 cluster reduces *Rankl* gene expression and does not alter *Opg* mRNA levels (see Fig. 5b). RANKL exist as membrane-bound and soluble protein and the former seems to be more functionally significant than the latter^32^. We hypothesized that cell surface presentation of RANKL and direct contact with myeloid progenitor cells may be crucial to induce osteoclastogenesis by chondrocytes. Coculture experiments of PECs isolated from newborn male Col2a1-Cre (Cre) and Col2a1-Cre-*Mirc24*^*tm1M/Y*^ (hKO) mice and myeloid progenitor populations derived from spleen^33^ indicate that indeed osteoclast numbers were reduced in PECs from newborn hKO mice compared to Cre control, but high variations between individual cocultures were detected owing to the presence of a high degree of nonhypertrophic chondrocytes in coculture experiments (see Supplementary Fig. S9 online).

Interestingly, neither *Rankl* nor *Opg* is a predicted target of the Mirc24 cluster-encoded miRNAs^34^. Moreover, miRNAs usually inhibit the expression of their target genes with only few miRNAs known to stabilize their mRNA targets^34^. Recently, we showed that miR-322 interacts with *Mek1* in a direct manner and stabilizes *Mek1* mRNA to increase MEK1 protein levels^15^. Thus, the increase in *Rankl* and *Opg* mRNA levels observed in miR-322 and miR-503 transfected chondrocytes is probably secondary to an interaction of cluster-encoded miRNAs with other targets. *Rankl* and *Opg* expression in chondrocytes was not induced by activation of the RAF/MEK/ERK signaling pathway, but was stimulated by FCS (see Supplementary Fig. S10 online). RANKL has been shown to stimulate *Ctsk* expression in a time- and dose-dependent manner^35–37^ and in Mirc24 cluster-depleted newborn mice lower *Rankl* mRNA levels result in a decreased *Ctsk* gene expression. Decreased *Rankl* expression may also cause a lower production and secretion of RANKL by hypertrophic chondrocytes. Secreted RANKL acts in a paracrine manner and diffuses over a short distance to reach its receptor RANK on the surface of osteoclast precursors, whereas membraneous RANKL acts in a juxtacrine manner and requires cell–cell contact to transduce signaling. Reduced release or presentation of RANKL by hypertrophic chondrocytes may therefore directly affect osteoclast progenitor differentiation and bone remodeling via blood vessel infiltration, since these cells are located in close association to the chondro-osseous junction^38^.

MiRNAs are considered to have a high clinical potential as disease-specific biomarkers or therapeutic agents to inhibit bone resorption. However, the complexity of gene regulation by miRNAs needs to be studied in greater depth. In this study we show that lack of the Mirc24 cluster in cartilage decreases *Rankl* gene expression in chondrocytes and that cluster-encoded miR-322 and miR-503 have the potential to modulate bone remodeling in vivo.

## Materials and methods

### Mice

MicroRNA cluster 24 transgenic mice (Mirc24^*tm1M/tm1M*^), which contain a LacZ reporter gene and lox sites to inactivate the cluster-encoded miR-322, miR-351 and miR-503^14^, Col2a1-Cre mice^13^ and C57BL/6N mice were used for animal studies. Male hemizygous knockout mice (hKO) Col2a1-Cre-Mirc24^tm1M/Y^ and Col2a1-Cre (Cre) littermate controls were generated as previously described^15^. All experimental protocols were performed with male mice in agreement with the guidelines of the German animal protection law and were approved by a licensing committee (Institutional Review Board: “Landesamt für Natur, Umwelt und Verbraucherschutz Nordrhein Westfalen”) #No. 4.20.028. The study complies with ARRIVE guidelines.

### Micro CT analysis

Femur morphology and architecture was evaluated using a high-resolution micro-CT scanner (μCT 35; Scanco Medical AG, Switzerland) as described previously^15^. Isolated femora of male newborn Cre and hKO mice were scanned with an isotropic voxel size of 3.5 µm using 45 kVp tube voltage, 115 µA tube current and 400 ms integration time. The raw images were preprocessed using a 3D Gaussian filter algorithm and the mineralized tissue was separated from soft tissues by a global thresholding procedure to remove image noise gray-scale data. The segmentation steps were applied with support = 1.0, sigma = 0.8. For analysis, the total length of the calcified primary ossification center (POC) of newborn femora was determined. Bone volume fraction (BV/TV) and tissue mineral density (TMD) were then calculated at 50% of this total length using a threshold of 26% of the maximum grey scale value. The volume of interest (VOI) covered 5% of the total length.

### Preparation of sections

Femora from male newborn Cre and hKO mice were isolated and either fixed in 4% (w/v) paraformaldehyde overnight, embedded in paraffin and sectioned using a microtome (HM355 S, Thermo Fisher Scientific, USA) or embedded in O.C.T. Tissue-Tek (Sakura Finetek, Tokyo, Japan) and sectioned using a Leica Cryotome CM3050.

### Fluorescence in situ hybridization analysis

Transcripts were detected on frozen sections of femora (7 µm, n = 4 biological replicates per genotype) isolated from male newborn Cre and hKO mice as described previously^16^ using the QuantiGene ViewRNA ISH Tissue Assay Kit and QuantiGene ViewRNA ISH Cell Assay Kit (Affymetrix, USA) according to the manufacturer’s instructions. Alexa Fluor 488 labeled probes (Affymetrix, USA) were used to detect *Col1a1* (NM_007742.3) and *Ctsk* (NM_007802.4) expression in situ. Sections were analyzed by immunofluorescence microscopy (Nikon Eclipse TE2000-U Microscope, Nikon, Tokyo, Japan). For quantification, the Ctsk^+^ pixels per mm^2^ was determined.

### Immunofluorescence analysis

Frozen sections of femora fixed with 4% (w/v) PFA for 10 min or deparaffinized sections (7 µm, n = 4 biological replicates per genotype) were pretreated with 5 mg/mL hyaluronidase (Sigma-Aldrich, Germany) for 30 min at 37 °C and with 10 µg/mL proteinase K (Qiagen, Germany) for 10 min at 55 °C. Sections were permeabilized with 0.25% (v/v) TritonX-100 in TBS for 10 min and incubated with 10% (v/v) FCS and 5% (v/v) normal goat serum blocking solution. Sections were incubated with a primary antibody specific for OPN (AKm2A1, 1/100, Santa Cruz Biotechnology, USA), collagen I (1/1000, Cedarlane, Canada) or collagen II (II-4C11, 1/500, Millipore, USA) and the corresponding secondary antibodies coupled to Cy3 (1/800, Jackson ImmunoResearch, USA) using standard protocols. Sections were mounted in mounting medium and analyzed by immunofluorescence microscopy (Nikon Eclipse TE2000-U Microscope).

### Collagen hybridizing peptide staining

Frozen sections of femora isolated from male newborn Cre and hKO mice (7 µm, n = 4 biological replicates per genotype) were fixed with 4% (w/v) PFA for 10 min and incubated with 5% (v/v) normal goat serum blocking solution. To prevent self-assembly of triple helices by collagen hybridizing peptide (R-CHP, 3Helix, USA), 5 µM CHP was heated at 80 °C for 5 min prior to staining and incubated for 1 min on ice to quench to room temperature. CHP was applied to the sections overnight at 4 °C and sections were mounted in mounting medium and analyzed by immunofluorescence microscopy (Nikon Eclipse TE2000-U Microscope).

### Immunohistochemistry analysis

Frozen sections of femora isolated from male newborn Cre and hKO mice (7 µm, n = 3 biological replicates per genotype) were fixed with 4% (w/v) PFA for 10 min, pretreated with citrate buffer (pH 6.0) for 1 h at 60 °C and incubated with an osterix-specific primary antibody (1/500, Abcam, UK). The corresponding secondary antibody labeled with horseradish peroxidase was used for detection (Zytomed Systems, Germany). Sections were mounted in Kaiser's glycerol gelatin and analyzed (Nikon Eclipse TE2000-U microscope).

### Toluidine Blue and Giemsa histological staining

To determine osteoblast numbers, deparaffinized sections (7 µm) were stained for Toluidine Blue (n = 6 biological replicates per genotype) and Giemsa (n = 4 biological replicates per genotype). For, sections were stained for 2 min at room temperature with Toluidine Blue (0.1% Toluidine Blue O, 1% sodium chloride, 7% ethanol, pH 2.3) or for 20 min at room temperature with Giemsa's azur eosin methylene blue solution (Sigma-Aldrich, USA). After washing and dehydration, sections were mounted in DPX (Sigma-Aldrich, USA) and analyzed by light microscopy (Axiophot fluorescence microscope, Zeiss, Germany). For quantification, the number of osteoblasts per mm trabecular bone was calculated.

### Histochemical assay of ALP activity

For analysis of ALP activity, frozen sections of femora isolated from male newborn Cre and hKO mice (7 µm, n = 4 biological replicates per genotype) were stained using the Leukocyte Alkaline Phosphatase Kit (Sigma-Aldrich, USA) according to the manufacturer’s instructions. Briefly, sections were fixed for 30 s, stained for 1 h at 37 °C, mounted in Kaiser's glycerol gelatin and analyzed by light microscopy (Axiophot fluorescence microscope, Zeiss, Germany).

### Histochemical assay of TRAP activity

For analysis of osteoclast activity, frozen sections of femora isolated from male newborn Cre and hKO mice (7 µm, n = 4 biological replicates per genotype) were stained using the Acid phosphatase, leukocyte (TRAP) Kit (Sigma-Aldrich, USA) according to the manufacturer’s instructions. Sections were fixed for 30 s, stained for 2 h at 37 °C, mounted in Kaiser's glycerol gelatin and analyzed by light microscopy (Axiophot fluorescence microscope, Zeiss, Germany). To determine osteoclast numbers, deparaffinized sections (7 µm, n = 4 biological replicates per genotype) were stained for TRAP activity as previously described^22^. Briefly, sections were incubated in 50 mM sodium l-tartrate, 0.1 M sodium acetate, 1.6 mM Fast Red Violet LB salt (Sigma-Aldrich, USA), 0.3 mM Naphthol AS-MX phosphate (Sigma-Aldrich, USA) and 0.5% (v/v) 2-ethoxyethanol (Sigma-Aldrich, USA), pH 5 for 45 min at 37 °C, counterstained with 0.02% (w/v) Fast Green for 30 s, mounted in Kaiser's glycerol gelatin and analyzed by light microscopy (Axiophot fluorescence microscope, Zeiss, Germany). For quantification, the number of multinucleated cells per mm trabecular bone was calculated.

### Cell culture

Primary epiphyseal chondrocytes (PECs) were isolated from epiphyseal cartilage of newborn C57BL/6N mice as described previously^15,39^. Briefly, cartilage was digested overnight with 1.61 U/mL Collagenase P (11213865001, Sigma-Aldrich, Germany) at 37 °C. Cells were passed through a 100 μm and a 40 µm cell strainer (BD Biosciences), plated out at 22 500 cells/cm^2^ and cultured for 6 days in DMEM/F12 (Gibco, Thermo Fisher Scientific, USA) supplemented with 10% (v/v) FCS, penicillin (100 units/mL, Biochrom, UK), streptomycin (100 μg/mL, Biochrom, UK), ascorbate (44 μg/mL, Sigma-Aldrich, Germany) and l-ascorbate-2-phosphate (130 μg/mL, Sigma-Aldrich, Germany) at 37 °C and 5% CO_2_. PECs isolated from male newborn Cre and hKO mice were cultured for 6 days, detached and cultured for 2 days for semi-quantitative PCR analysis.

### Chondrocyte/spleen cell co-culture assay

PECs were isolated from newborn Mirc24 mice, cultured for 6 days, detached and 3 × 10^3^ cells were seeded on glass chamber slides (Nunc™ Lab-Tek™ II, Thermo Fisher Scientific, USA). Spleen cells were isolated from 2 months old C57BL/6N mice, passed through a 40 µm cell strainer and subsequent lysed in erythrocyte lysis buffer for 10 min on ice. 3 × 10^5^ spleen cells were seeded into the culture and co-cultured with the chondrocytes for 3 days in minimum essential medium-α (α-MEM) supplemented with 10% FCS and 30 ng/mL M-CSF (Miltenyi Biotec, Germany). Cells were then treated with 30 ng/mL M-CSF and 10^−8^ M 1,25-(OH)_2_ vitamin D_3_ (Sigma-Aldrich, Germany) for additional 5 days. Cells were fixed for 10 min with 10% PFA, stained for 1 h 30 min at 37 °C for TRAP.

### RNA isolation and PCR analysis

Total RNA from primary epiphyseal chondrocytes was isolated by phenol–chloroform extraction using TRIzol reagent (Thermo Fisher Scientific, USA) and reversely transcribed into cDNA (Omniscript RT assay and miScript II RT assay, QIAGEN, Germany). 25 ng cDNA was used for semi-quantitative PCR assays (REDTaq ReadyMix™, Sigma-Aldrich, Germany). The gene expression was normalized to β-actin (*Actb*). The following primers were used: *Actb* (forward: 5′-GACGAGGCCCAGAGCAAGAG-3′; reverse: 5′-CTAGAGCAACATAGCACAGC-3′), *Opg* (forward: 5′-GGAAGACCATGAGGTTCCTG-3′; reverse: 5′-CCTTCATCTAGGGGTGACAC-3′) and *Rankl* (forward: 5′-CCTGAGACTCCATGAAAACGC-3′; reverse: 5′-CCCGATGTTTCATGATGCCG-3′). 10 ng cDNA was used for quantitative PCR assays (miScript SYBR Green PCR Kit, QIAGEN, Germany)^15^. The expression of miR-322 and miR-503 was normalized to RNU6B and the fold change was determined by the delta-delta C_t_ method^40^.

### Genotyping analysis

Genomic DNA was analyzed by PCR using the following primers for detecting the Mirc24 locus in a genotyping PCR (wildtype allele: 488 bp, mutant allele: 580 bp) and deletion PCR (wildtype allele: 4278 bp, mutant allele: 678 bp):

- miR322 tm1Mtm.fw 5′-ACACACACCCTT CTGGTCTG-3′
- miR322 tm1Mtm.rv 5′-CGCTCTTCCTCTGGCATAAG-3′
- mirc24.floxp 5′-CGCTACCATTACCAGTTGGT-3′

### Approval for animal experiments

All experimental protocols were performed in accordance with the animal ethics guidelines of the German animal protection law and were approved by a licensing committee (Institutional review board—“Landesamt für Natur, Umwelt und Verbraucherschutz Nordrhein-Westfalen”) #No. 4.20.028. The study complies with the ARRIVE guidelines.

## Supplementary Information

Supplementary Figures.

## References

[1] Papaioannou G (2015). miRNAs in bone development. Curr. Genomics.

[2] Zhou X (2014). Chondrocytes transdifferentiate into osteoblasts in endochondral bone during development, postnatal growth and fracture healing in mice. PLoS Genet..

[3] Yang L, Tsang KY, Tang HC, Chan D, Cheah KS (2014). Hypertrophic chondrocytes can become osteoblasts and osteocytes in endochondral bone formation. Proc. Natl. Acad. Sci. U.S.A..

[4] Long JT (2022). Hypertrophic chondrocytes serve as a reservoir for marrow-associated skeletal stem and progenitor cells, osteoblasts, and adipocytes during skeletal development. Elife.

[5] Gibson G (1998). Active role of chondrocyte apoptosis in endochondral ossification. Microsc. Res. Tech..

[6] Mackie EJ, Ahmed YA, Tatarczuch L, Chen KS, Mirams M (2008). Endochondral ossification: How cartilage is converted into bone in the developing skeleton. Int. J. Biochem. Cell Biol..

[7] Gradus B, Alon I, Hornstein E (2011). miRNAs control tracheal chondrocyte differentiation. Dev. Biol..

[8] Kobayashi T (2008). Dicer-dependent pathways regulate chondrocyte proliferation and differentiation. Proc. Natl. Acad. Sci. U.S.A..

[9] Kobayashi T (2015). Early postnatal ablation of the microRNA-processing enzyme, Drosha, causes chondrocyte death and impairs the structural integrity of the articular cartilage. Osteoarthr. Cartil..

[10] Papaioannou G, Inloes JB, Nakamura Y, Paltrinieri E, Kobayashi T (2013). let-7 and miR-140 microRNAs coordinately regulate skeletal development. Proc. Natl. Acad. Sci..

[11] Gaur T (2010). Dicer inactivation in osteoprogenitor cells compromises fetal survival and bone formation, while excision in differentiated osteoblasts increases bone mass in the adult mouse. Dev. Biol..

[12] Mizoguchi F (2010). Osteoclast-specific Dicer gene deficiency suppresses osteoclastic bone resorption. J. Cell. Biochem..

[13] Ovchinnikov DA, Deng JM, Ogunrinu G, Behringer RR (2000). Col2a1-directed expression of Cre recombinase in differentiating chondrocytes in transgenic mice. Genesis.

[14] Park CY (2012). A resource for the conditional ablation of microRNAs in the mouse. Cell Rep..

[15] Bluhm B (2017). miR-322 stabilizes MEK1 expression to inhibit RAF/MEK/ERK pathway activation in cartilage. Development.

[16] Georgieva VS (2020). Ablation of the miRNA cluster 24 has profound effects on extracellular matrix protein abundance in cartilage. Int. J. Mol. Sci..

[17] Holzer T (2019). Respiratory chain inactivation links cartilage-mediated growth retardation to mitochondrial diseases. J. Cell Biol..

[18] Chung HK (2017). Growth differentiation factor 15 is a myomitokine governing systemic energy homeostasis. J. Cell Biol..

[19] Sommer B, Bickel M, Hofstetter W, Wetterwald A (1996). Expression of matrix proteins during the development of mineralized tissues. Bone.

[20] Andersson G (2003). TRACP as an osteopontin phosphatase. J. Bone Miner. Res..

[21] Ek-Rylander B, Andersson G (2010). Osteoclast migration on phosphorylated osteopontin is regulated by endogenous tartrate-resistant acid phosphatase. Exp. Cell Res..

[22] Heilig J (2020). Collagen IX deficiency leads to premature vascularization and ossification of murine femoral heads through an imbalance of pro- and antiangiogenic factors. Osteoarthr. Cartil..

[23] Fonović M, Turk B (2014). Cysteine cathepsins and extracellular matrix degradation. Biochim. Biophys. Acta (BBA) Gen. Subj..

[24] Kishimoto K, Kitazawa R, Kurosaka M, Maeda S, Kitazawa S (2006). Expression profile of genes related to osteoclastogenesis in mouse growth plate and articular cartilage. Histochem. Cell Biol..

[25] Boskey AL (2007). Handbook of Biomineralization.

[26] Gamez B, Rodriguez-Carballo E, Bartrons R, Rosa JL, Ventura F (2013). MicroRNA-322 (miR-322) and its target protein Tob2 modulate Osterix (Osx) mRNA stability. J. Biol. Chem..

[27] Chen C (2014). MiR-503 regulates osteoclastogenesis via targeting RANK. J. Bone Miner. Res..

[28] Couasnay G, Madel MB, Lim J, Lee B, Elefteriou F (2021). Sites of Cre-recombinase activity in mouse lines targeting skeletal cells. J. Bone Miner. Res..

[29] Wang W (2011). Mice lacking Nf1 in osteochondroprogenitor cells display skeletal dysplasia similar to patients with neurofibromatosis type I. Hum. Mol. Genet..

[30] Kafienah W, Brömme D, Buttle DJ, Croucher LJ, Hollander AP (1998). Human cathepsin K cleaves native type I and II collagens at the N-terminal end of the triple helix. Biochem. J..

[31] Xiong J (2011). Matrix-embedded cells control osteoclast formation. Nat. Med..

[32] Ono T, Hayashi M, Sasaki F, Nakashima T (2020). RANKL biology: Bone metabolism, the immune system, and beyond. Inflamm. Regen..

[33] Wang B, Jin H, Shu B, Mira RR, Chen D (2015). Chondrocytes-specific expression of osteoprotegerin modulates osteoclast formation in metaphyseal bone. Sci. Rep..

[34] Agarwal V, Bell GW, Nam JW, Bartel DP (2015). Predicting effective microRNA target sites in mammalian mRNAs. Elife.

[35] Shalhoub V (1999). Osteoprotegerin and osteoprotegerin ligand effects on osteoclast formation from human peripheral blood mononuclear cell precursors. J. Cell. Biochem..

[36] Corisdeo S, Gyda M, Zaidi M, Moonga BS, Troen BR (2001). New insights into the regulation of cathepsin K gene expression by osteoprotegerin ligand. Biochem. Biophys. Res. Commun..

[37] Pang M, Martinez AF, Jacobs J, Balkan W, Troen BR (2005). RANK ligand and interferon gamma differentially regulate cathepsin gene expression in pre-osteoclastic cells. Biochem. Biophys. Res. Commun..

[38] Gerber HP (1999). VEGF couples hypertrophic cartilage remodeling, ossification and angiogenesis during endochondral bone formation. Nat. Med..

[39] Belluoccio D (2010). Sorting of growth plate chondrocytes allows the isolation and characterization of cells of a defined differentiation status. J. Bone Miner. Res..

[40] Pfaffl MW (2001). A new mathematical model for relative quantification in real-time RT-PCR. Nucleic Acids Res..

