# Supplementary material for: Ablation of the miRNA cluster 24 in cartilage and osteoblasts impairs bone remodeling
Source: Sci Rep. 2022 Jun 1;12:9116. doi: 10.1038/s41598-022-13231-z (PMC9160244; doi:10.1038/s41598-022-13231-z)
Supplement: Supplementary file 1 — Supplementary Figures. [file 41598_2022_13231_MOESM1_ESM.pdf]

Supplementary Information

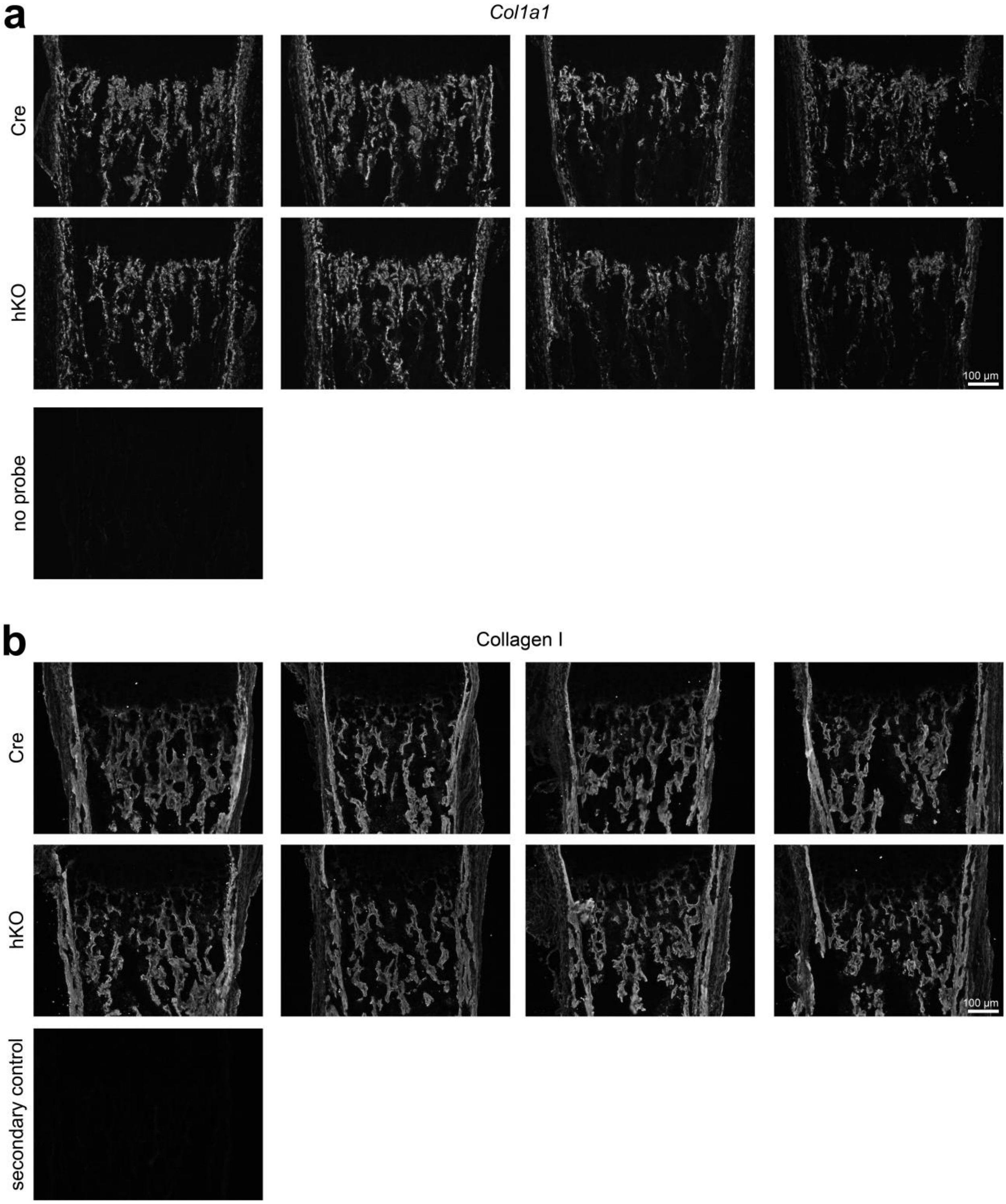

Supplementary Figure S1. Characterization of collagen I gene expression and protein abundance in femoral sections of Mirc24 cluster-deficient mice. Assays were performed in plane matched sections of femora from newborn Col2a1-Cre (Cre) and Col2a1-Cre-*Mirc24*$^{tm1M/Y}$ (hKO) mice (n=4 per genotype). (**a**) *Col1a1* mRNA distribution was analyzed by fluorescence *in situ* hybridization using a *Col1a1* specific fluorescence probe. Control without fluorescence probe is shown. (**b**) The abundance of collagen I was studied by immunofluorescence analysis. A secondary antibody control is shown. Brightness was adjusted for visualization (**a**, **b**).

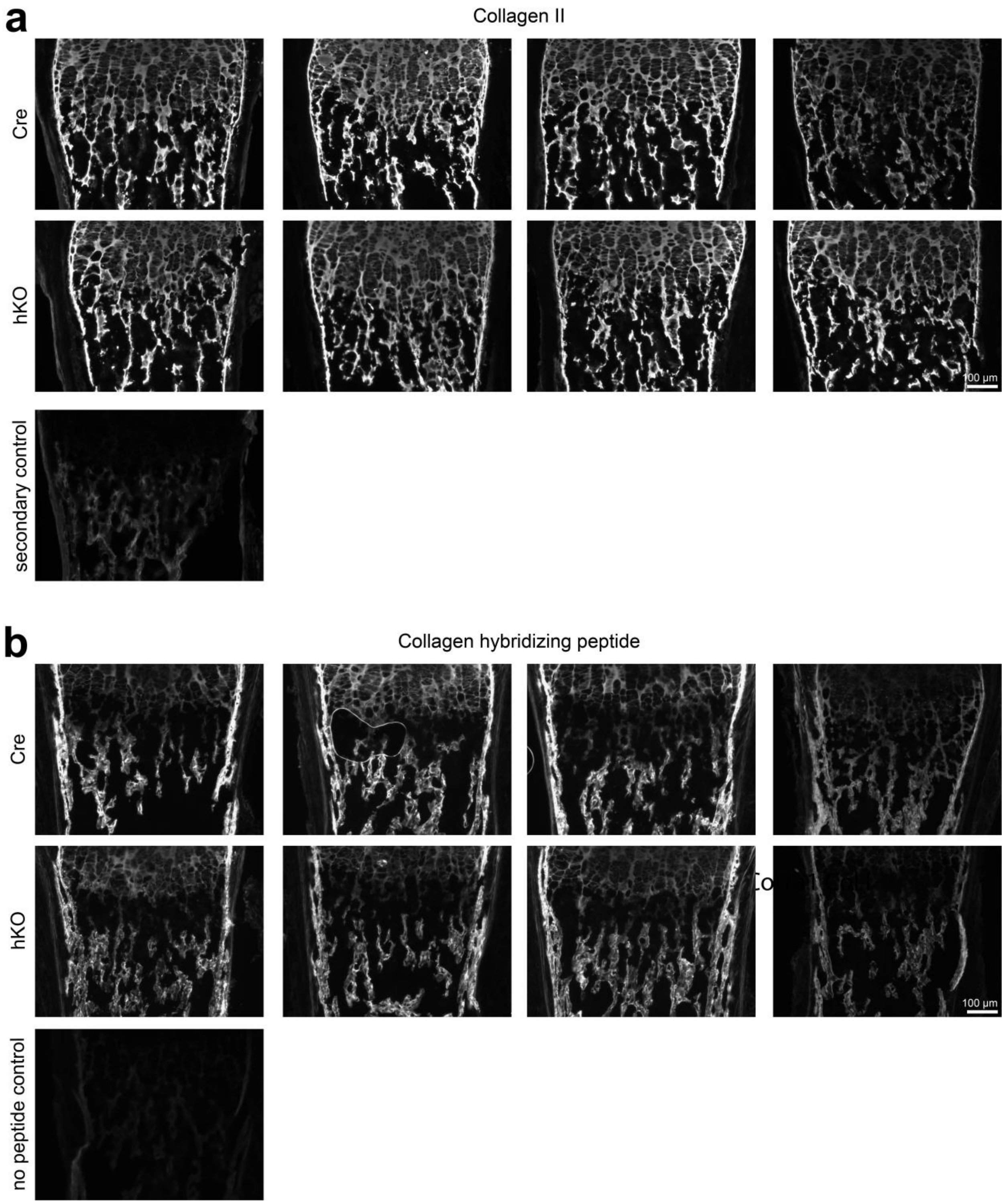

Supplementary Figure S2. Characterization of collagen II protein abundance and collagen remodeling in femoral sections of Mirc24 cluster-deficient mice. Stainings were performed in plane matched sections of femora from newborn Col2a1-Cre (Cre) and Col2a1-Cre-*Mirc24*$^{tm1M/Y}$ (hKO) mice (n=4 per genotype). (**a**) The abundance of collagen II was studied by immunofluorescence analysis. A secondary antibody control is shown. (**b**) To study bone remodeling the binding of collagen hybridizing peptide to unfolded collagen chains was analyzed by fluorescence microscopy. A control without collagen hybridization peptide is shown. Brightness was adjusted for visualization (**a**).

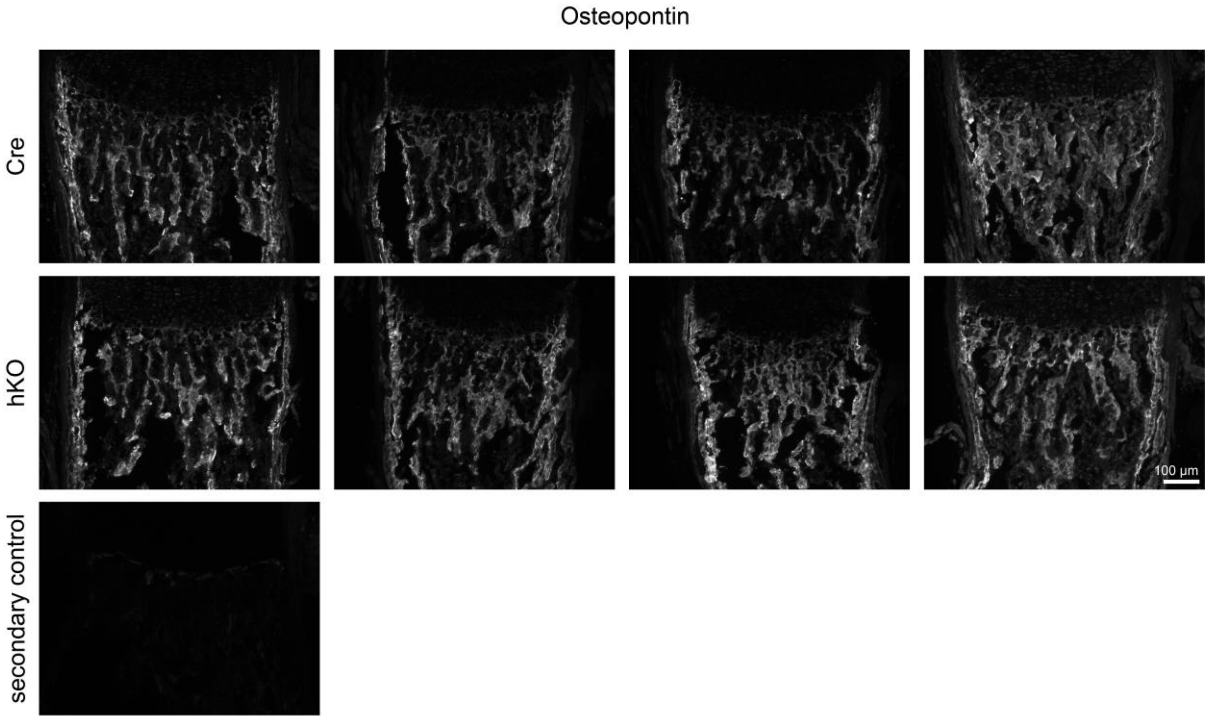

Supplementary Figure S3. Characterization of osteopontin distribution in femoral sections from Mirc24 cluster-deficient mice. Stainings were performed in plane matched sections of femora from newborn Col2a1-Cre (Cre) and Col2a1-Cre-*Mirc24*$^{tm1M/Y}$ (hKO) mice (n=4 per genotype). A secondary antibody control is shown.

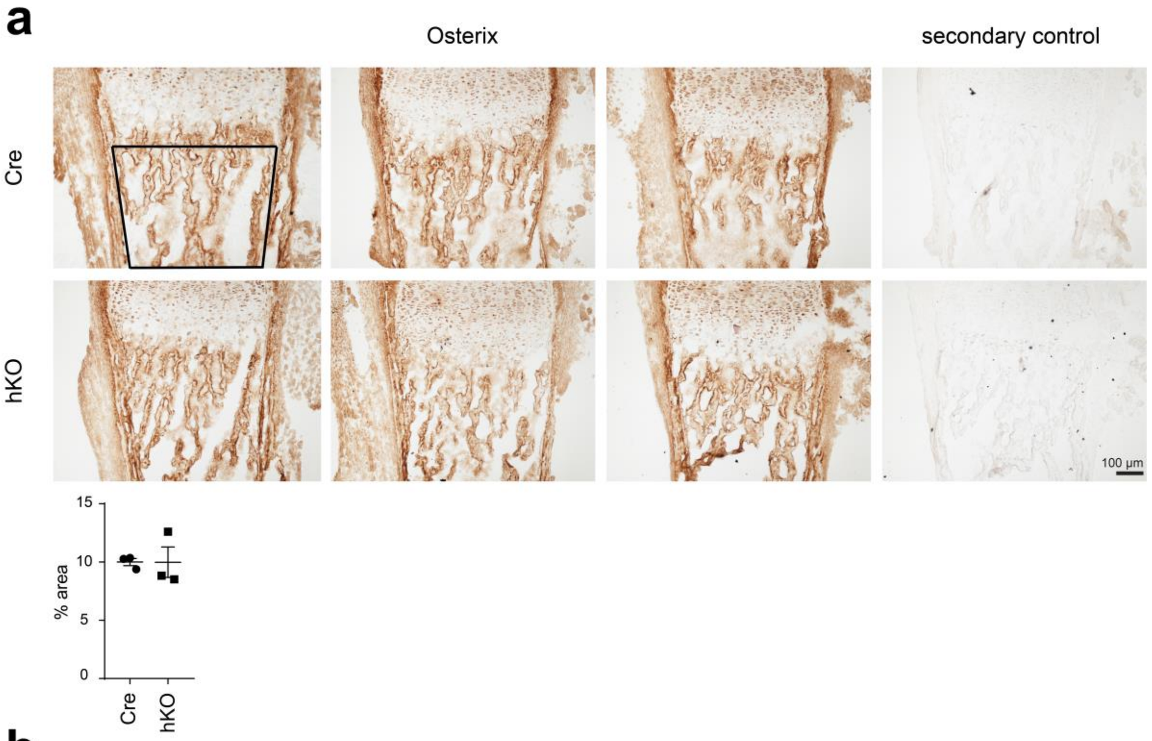

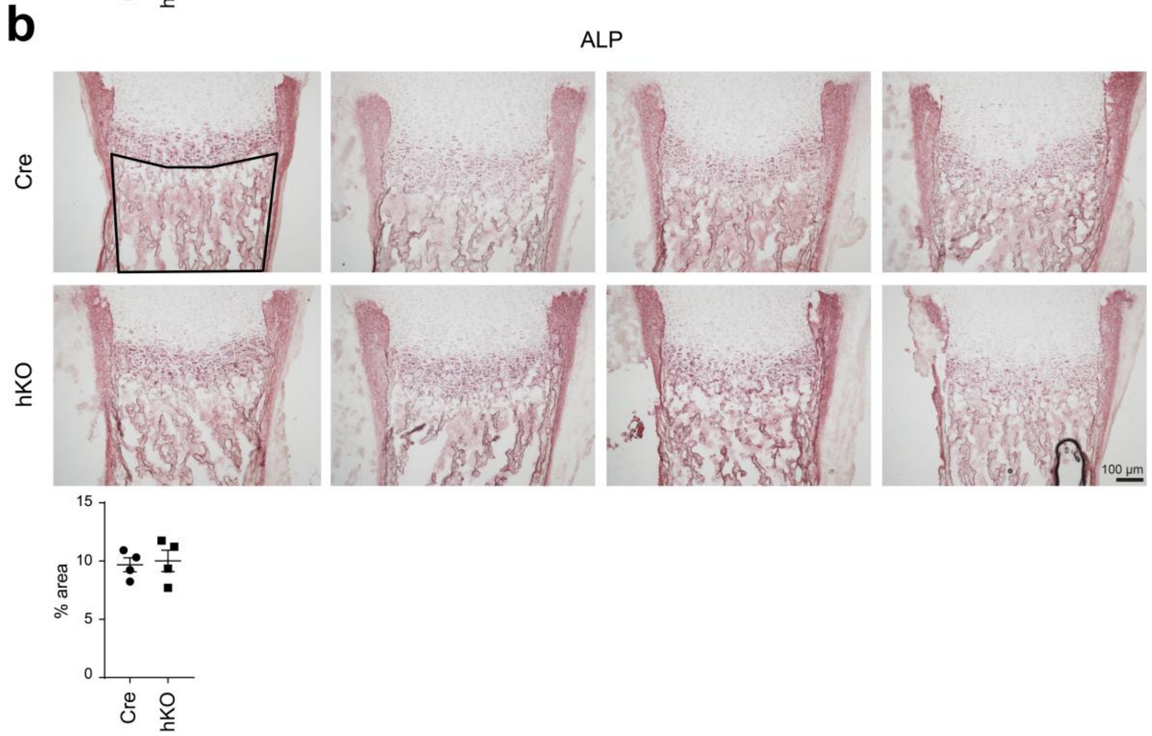

Supplementary Figure S4. Characterization of osterix localization and ALP activity in femoral sections from Mirc24 cluster-deficient mice. Stainings were performed in plane matched sections of femora from newborn Col2a1-Cre (Cre) and Col2a1-Cre-*Mirc24*$^{tm1M/Y}$ (hKO) mice. (**a**) Osterix distribution was analyzed by immunostaining (n=3 per genotype). A secondary antibody control is shown. (**b**) Activity of alkaline phosphatase (ALP) was detected by histological staining (n=4 per genotype). Image J quantification was used to determine the percentage of stained area in the region of interest (box) in section from Cre and hKO mice.

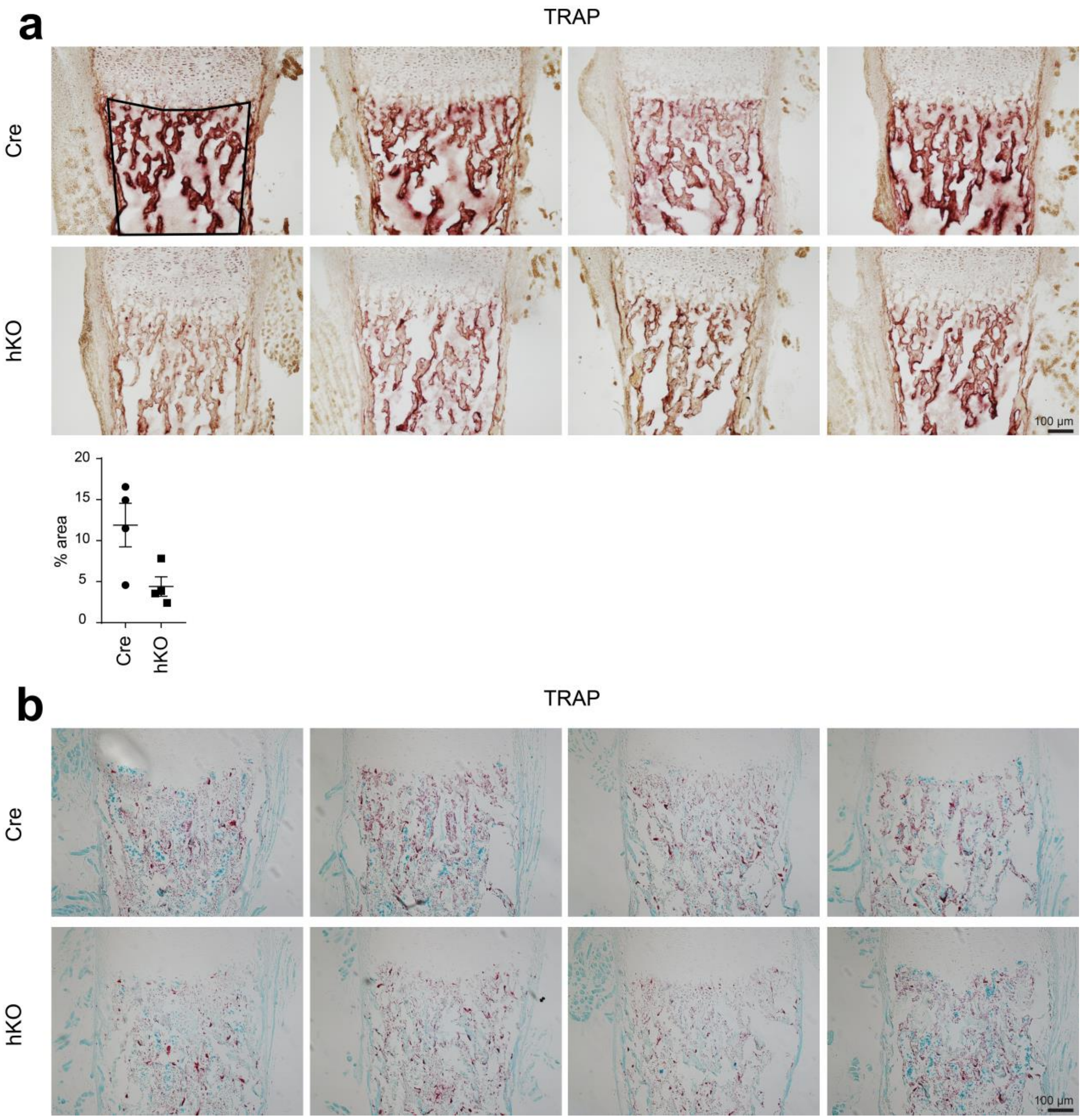

Supplementary Figure S5. Characterization of TRAP activity in femoral sections from Mirc24 cluster-deficient mice. (**a**) Tartrate-resistant acid phosphatase (TRAP) activity stainings were performed in frozen sections (n=4 per genotype). Image J quantification was used to determine the percentage of stained area in the region of interest (box) in section from Cre and hKO mice. TRAP activity (red) was detected on paraffin sections counterstained by Fast green (blue, n=4 per genotype). Brightness was adjusted for visualization (**b**).

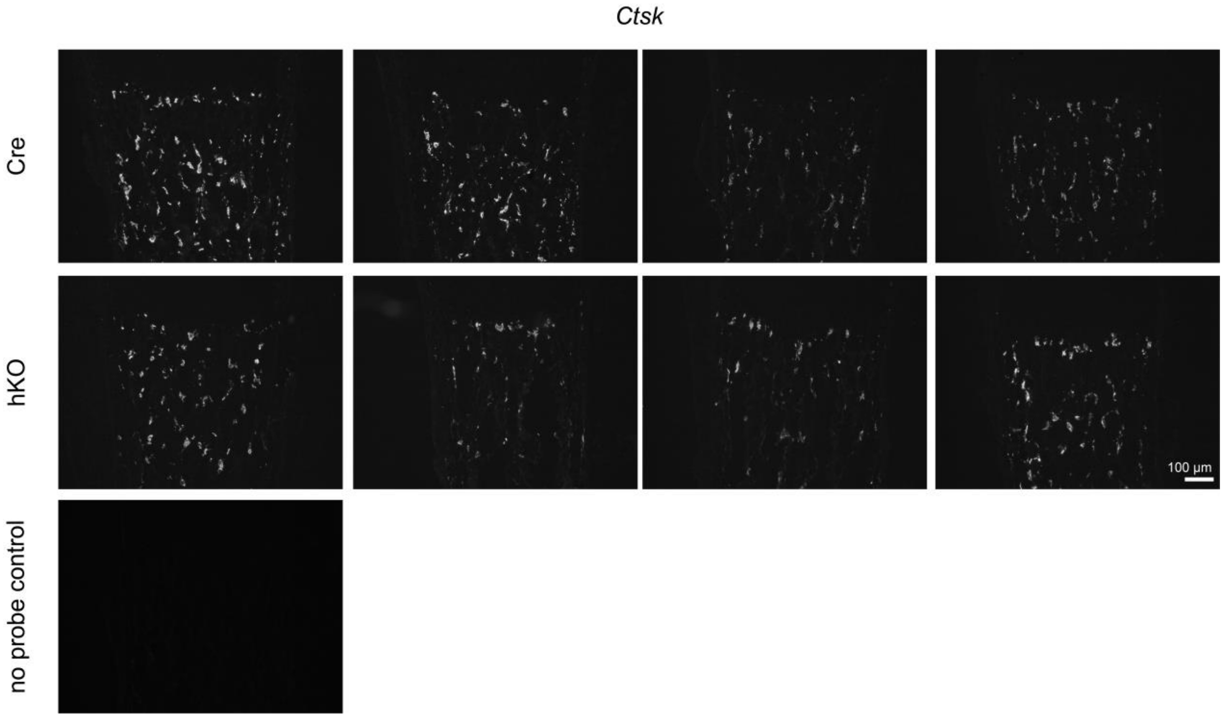

Supplementary Figure S6. Characterization of *Ctsk* expression in femoral sections from Mirc24 cluster-deficient mice. *Ctsk* mRNA distribution was analyzed by fluorescence *in situ* hybridization using a *Ctsk* specific fluorescence probe (n=4 per genotype). A control without fluorescence probe is shown. Brightness was adjusted for visualization.

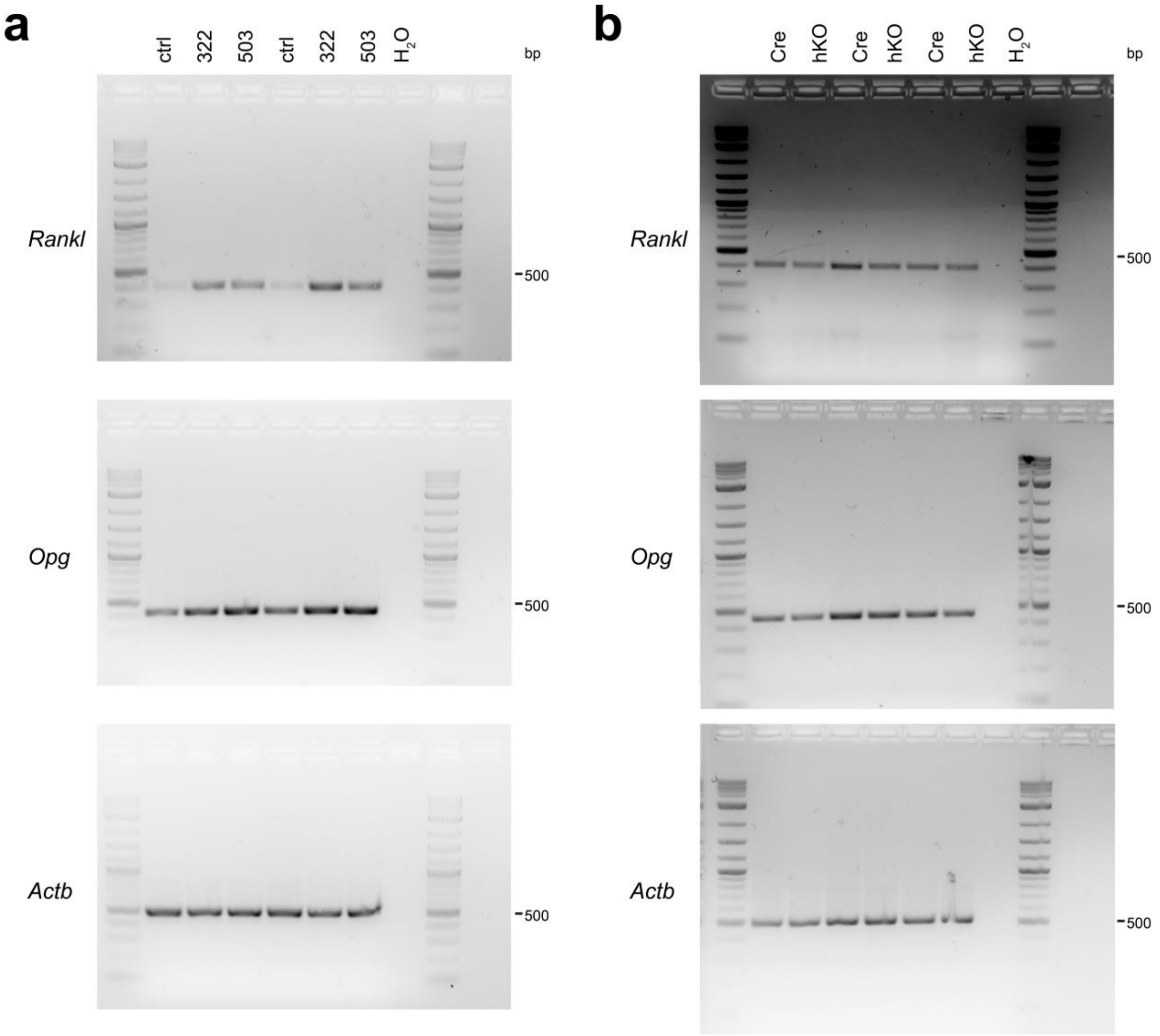

Supplementary Figure S7. Original gels of Figure 5.

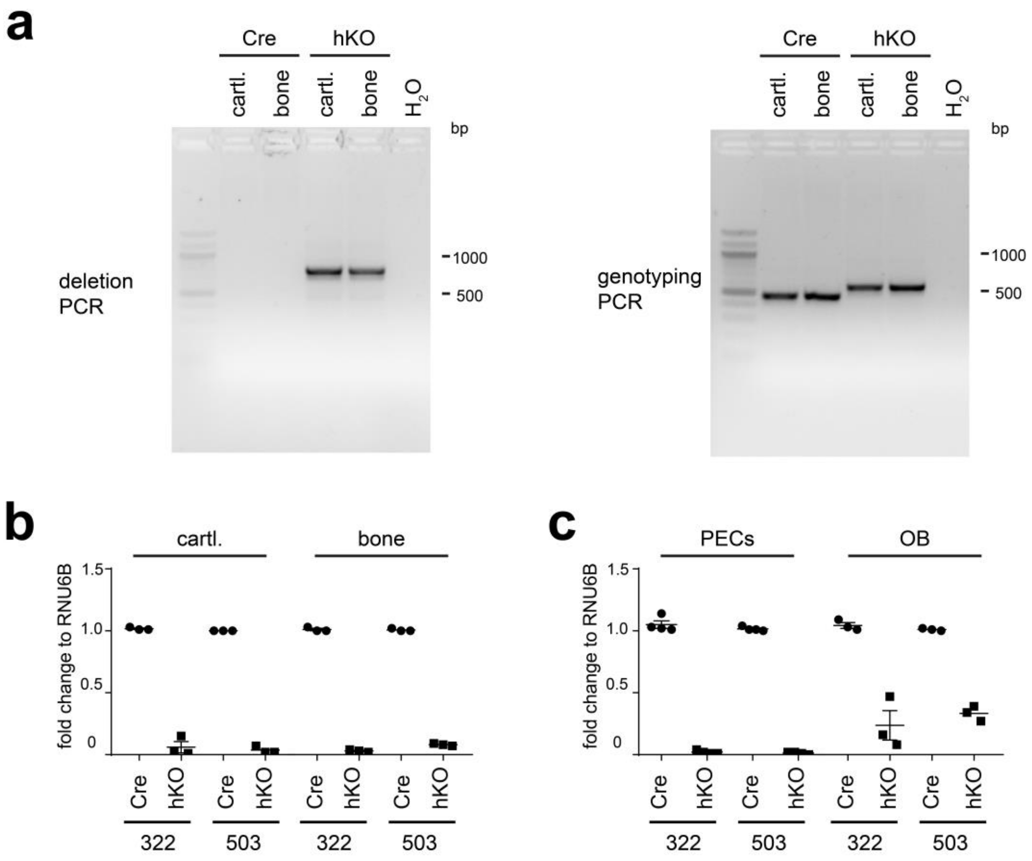

Supplementary Figure S8. (a) PCR analysis of cartilage-specific cluster deletion in isolated cartilage and bone from Col2a1-Cre (Cre) and Col2a1-Cre-*Mirc24*$^{tm1M/Y}$ (hKO) mice. (b, c) The relative expression changes of miR-322 and miR-503 in tissue and isolated cells was determined by quantitative PCR analysis. PECs - primary epiphyseal chondrocytes, OB – osteoblasts.

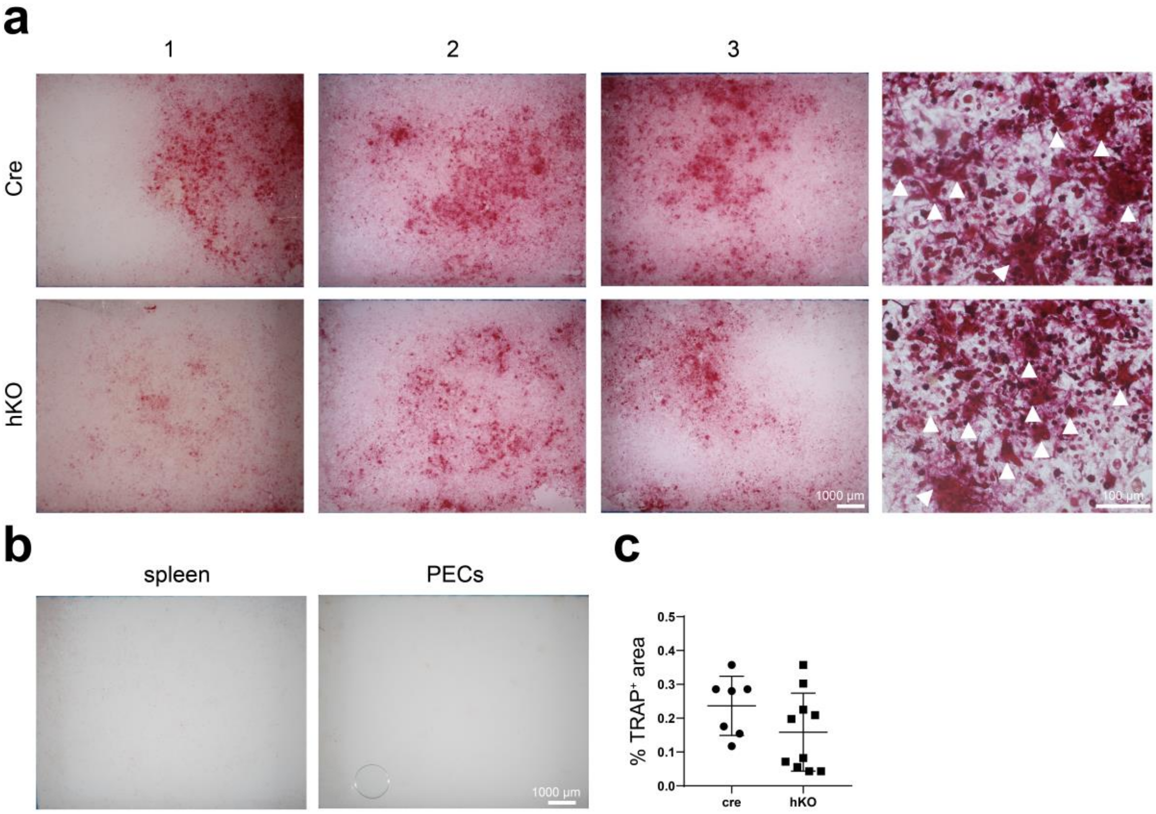

Supplementary Figure S9. Characterization of chondrocytes-dependent osteoclast formation. (a) Spleen cells were isolated from two months old wild type female mice and were co-cultured with PECs isolated from newborn Col2a1-Cre (Cre) and Col2a1-Cre-*Mirc24*$^{tm1M/Y}$ (hKO) mice in the presence of 30 ng/mL M-CSF for three days, followed by cultivation in the presence of 30 ng/mL M-CSF and 10^{-8} M VitD3 for additional five days. Osteoclasts formation was determined by TRAP staining. Three representative images are shown per genotype and a close up is provided. Multinucleated osteoclasts are marked by arrowheads (close up). (b) Monocultures of spleen cells or PECs showed no positive TRAP staining. Scale: (a, b) 1000 µm, (a, close up) 100 µm. (c) The percentage of $TRAP^{+}$ area was determined by ImageJ analysis for cocultures originating from 7 Cre and 10 hKO mice.

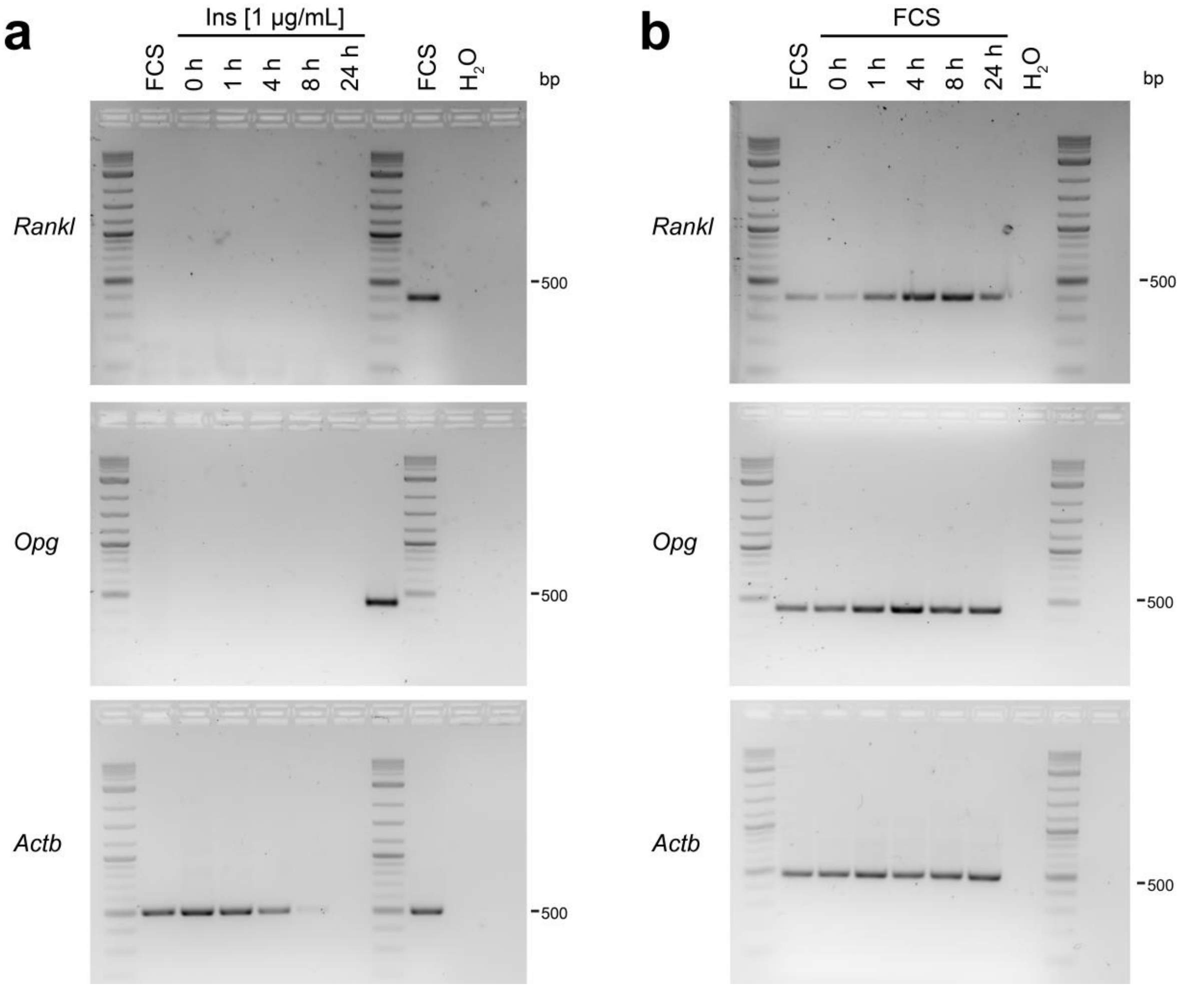

Supplementary Figure S10. Determination of *Rankl* and *Opg* gene expression upon insulin and FCS stimulation. The expression of *Rankl* and *Opg* was analyzed by semi-quantitative PCR in wild type PECs, which were serum starved for 24 h and stimulated with (**a**) 1 µg/mL insulin or (**b**) FCS for up to 24 h. The control (H_2O) contains no genomic DNA, FCS treated cells served as a positive control (FCS) and *Actb* as a loading control. Size of NEB Quick-Load® Purple 1 kb Plus DNA Ladder bands is given.
